# Efficacy of turmeric in the treatment of digestive disorders: a systematic review and meta-analysis protocol

**DOI:** 10.1186/2046-4053-3-71

**Published:** 2014-06-28

**Authors:** Kednapa Thavorn, Muhammad M Mamdani, Sharon E Straus

**Affiliations:** 1 Li Ka Shing Knowledge Institute, St. Michael’s Hospital, M5B 1W8 Toronto, ON, Canada; 2 Institute for Clinical Evaluative Sciences, M4N 3M5 Toronto, ON, Canada; 3 Institute of Health Policy, Management, and Evaluation, University of Toronto, M5T 3M6 Toronto, ON, Canada; 4 Leslie Dan Faculty of Pharmacy, University of Toronto, M5S 3M2 Toronto, ON, Canada; 5 Department of Geriatric Medicine, University of Toronto, M5S 1A8 Toronto, ON, Canada

## Abstract

**Background:**

Digestive disorders pose significant burdens to millions of people worldwide in terms of morbidity, mortality and healthcare costs. Turmeric has been traditionally used for conditions associated with the digestive system, and its therapeutic benefits were also confirmed in clinical studies. However, rigorous systematic review on this topic is severely limited. Our study aims to systematically review the therapeutic and adverse effects of turmeric and its compounds on digestive disorders, including dyspepsia, peptic ulcer, irritable bowel disease, Crohn’s disease, ulcerative colitis, and gastroesophageal reflux disease.

**Methods/Design:**

This study will include both randomized controlled trials and non-randomized controlled trials assessing the efficacy and safety of turmeric or its compounds in comparison to a placebo or any other active interventions for digestive disorders without any restrictions on participant age or language of publication. The primary outcome is the proportion of patients that have experienced treatment success. Secondary outcomes are the prevalence of an individual symptom of digestive disorders, the proportion of patients who experienced relapse, the number of physician visits/hospitalization due to digestive disorders, health-related quality of life and the proportion of patients who experienced adverse events. Relevant studies will be identified through MEDLINE, EMBASE, AMED, Dissertations & Theses Database and the Cochrane Central Register of Control Trials from their inception to August 31, 2013. In addition, grey literature such as information published on drug regulatory agencies websites and abstracts/proceedings from conferences will also be reviewed. A calibration exercise will be conducted in a process of study screening, whereby two reviewers will independently screen titles and abstracts from the literature search. Any conflicts will be resolved through a subsequent team discussion. The same process will be adopted in data abstraction and methodological quality appraisal by the Cochrane Risk of Bias Tool and the Newcastle-Ottawa Scale. We will describe study and patient characteristics, risk of bias/methodological quality results, and outcomes of the included studies. If we have sufficient data and homogeneity, a random effects meta-analysis will be performed.

**Discussion:**

Our results will help patients and healthcare practitioners to make informed decisions when considering turmeric as an alternative therapy for digestive disorders.

**Trial registration:**

PROSPERO registry number: CRD42013005739.

## Background

Digestive disorders, including dyspepsia, peptic ulcer disease, irritable bowel disease (IBS), inflammatory bowel disease (IBD), and gastroesophageal reflux disease (GERD), affect millions of people worldwide and place a highly significant economic burden on the healthcare systems [1,2]. In the United States, the total cost of digestive disorders was approximately $142 billion in 2009 [3], and digestive cancers ($24.1 billion), liver disease ($13.1 billion), and GERD ($12.6 billion) were identified as the three most costly conditions. In the United Kingdom, total cost attributable to gastrointestinal (GI) diseases was approximately £8 billion in 1997 [4].

Digestive disorders are conventionally treated with drugs and surgery, as well as psychological and behavioral therapy. Recently, alternative or complementary medicines, such as acupuncture, and herbal/dietary therapy, have become increasingly popular in persons with digestive disorders, especially when conventional therapies fail to improve their symptoms [5]. A survey of 539 patients attending an outpatient clinic in Spain showed that nearly two-thirds (61.6%) of patients with digestive disorders had used herbal therapies in the past year, and patients who were female, had a university education, or were diagnosed with lower GI disorders were found to be more frequent users of herbal therapies. Moreover, approximately 80% of these users were satisfied with the results these therapies yielded [6].

Turmeric is traditionally used as a herbal remedy for a variety of diseases in India and China and as an over-the-counter supplement worldwide [7]. Over the years, pre-clinical and clinical studies have shown numerous potential therapeutic activities, including anti-inflammatory [8], antioxidant [9], antimicrobial [10], antiplatelet [11], and anticancer effects [12], as well as choleretic and carminative actions [13]. In pre-clinical trials, turmeric was shown to potentially protect the GI tract through its anti-inflammatory effect. It also demonstrated its ability to increase the secretion of gastrin, secretin, and bicarbonate, gastric wall mucus and pancreatic enzyme [7], while inhibiting intestinal spasms and ulcer formation caused by stress, alcohol, indomethacin, pyloric ligation, and reserpine [14]. Turmeric was found to effectively improve dyspeptic symptoms in patients with dyspepsia, as well as maintain remission in patients with ulcerative colitis (UC); however, it did not significantly improve IBS-related outcomes. In particular, a randomized controlled trial (RCT) was conducted in 116 patients with dyspeptic complaints (such as abdominal pain, epigastric discomfort, flatulence or belching) [15]. The study findings indicate that after 7 days, 87% of the turmeric group experienced symptom relief from dyspepsia compared to 53% of the placebo group (*P* = 0.003). Another RCT was conducted to assess the effect of curcumin (the main active ingredient of turmeric) in 82 patients with UC [16]. These patients were randomly allocated to receive 1 g curcumin twice daily in addition to sulfasalazine or mesalamine or placebo as well as sulfasalazine or mesalamine for 6 months. At the end of the study period, fewer patients in the curcumin group experienced relapse compared to the control group (4.7% vs 20.5%, *P* = 0.038). In contrast, in a RCT of 106 patients with IBS, a consumption of 60 mg turmeric daily was found to have no significant therapeutic benefit over placebo in decreasing IBS-related pain and distention scores, other IBS symptoms, and psychological stress due to IBS [17].

To date, only one methodologically rigorous systematic review of the effects of turmeric (AMSTAR [18] score = 6) was conducted [19]. In this study, the authors reviewed the efficacy and safety of turmeric for maintenance remission in patients with UC. The findings suggest that, of 216 identified studies, only one study met the inclusion criteria, and this included study had a low risk of bias. This systematic review concluded that curcumin was a safe and effective therapy for maintenance of remission in patients with UC, in particular when supplement by mesalamine or sulfalazine.

Given the paucity of work on the therapeutic role of turmeric, we propose to complete a systematic review to determine the efficacy and safety of turmeric and its compounds in patients living with digestive disorders, including dyspepsia, peptic ulcer, IBS, IBD (Crohn’s disease and UC), and GERD. Our specific review questions are: 1) in patients with digestive disorders (that is, dyspepsia, peptic ulcer, IBS, IBD, or GERD), what are the effects of turmeric and its compounds on the treatment success, relapse rates, physician and hospitalization visits due to digestive disorders, and health-related quality of life; and 2) what adverse events are associated with turmeric and its compounds?

## Methods/Design

The systematic review protocol was designed based on the guidance from the PRISMA-P (Preferred Reporting Items for Systematic reviews and Meta-analyses Protocol) [20].

### Eligibility criteria

We will include studies of participants of any age, including children (<18 years old), and adults (≥18 years old), who were diagnosed with the following digestive disorders: dyspepsia, peptic ulcer, IBS, IBD (including Crohn’s disease and UC), and GERD. Dyspepsia is a group of symptoms that may include post-prandial fullness or an unpleasant sensation, such as prolonged persistence of food in the stomach, a feeling that the stomach is overfilled soon after starting to eat, epigastric pain, and epigastric burning [21]. A peptic ulcer is a defect in the lining of the stomach (gastric ulcer) or the first part of the small intestine (duodenal ulcer) [22]. Diagnosis is based on endoscopy by gastroenterologists. IBS is a functional GI disorder or a condition caused by changes in GI function. The most common symptoms of IBS are abdominal pain or discomfort, abdominal cramping, typically accompanied by diarrhea and/or constipation. Diagnosis is based on the Rome III diagnostic criteria for IBS [23]. IBD can be categorized into two diseases: Crohn’s disease and UC. Examples of the symptoms consist of diarrhea, constipation, pain or rectal bleeding with bowel movement, as well as abdominal pain/cramping. The condition is typically diagnosed by colonoscopy. GERD is defined as a collection of symptoms or tissue damage as a result of reflux of gastric contents into the esophagus [24]. Common symptoms include epigastric pain/discomfort, heartburn and regurgitation. GERD is usually diagnosed based on the symptoms and endoscopy may be required in some cases.

In this review, we will include studies in which patients in the treatment group received turmeric at any dosage regimen, formulation, and duration compared to a control group that received a placebo, no treatment, or any other active interventions for their GI condition. We will include experimental studies (RCTs, quasi-RCTs, controlled clinical trials), quasi-experimental studies (interrupted time series, controlled before after studies), and observational studies (cohort and case–control studies). We chose to include non-RCTs and observational studies because, in our scoping searches, we found few RCTs that examined the efficacy and safety of turmeric in patients with digestive disorders.

The primary outcome of interest is the proportion of patients who experienced treatment success (as defined by included studies, shown in Table 1). Secondary outcomes consist of prevalence of an individual symptom associated with digestive disorders (such as abdominal pain, epigastric pain), proportion of patients who experienced relapse (as defined by the included studies), the number of physician visits due to digestive disorders, the number of hospitalization visits due to digestive disorders, disease-specific quality of life, general health-related quality of life, and the proportion of patients who experienced adverse events.

**Table 1:** Examples of treatment success of gastrointestinal disorders

**Gastrointestinal disorder**	**Treatment success**
Dyspepsia	Improvement in dyspepsia clinical scores, such as a dyspepsia score [25,26], the Global Overall Severity score [27]
Peptic ulcer	• Complete healing of the ulcer as observed from endoscopy [28]; or
	• Presence of negative tests for *Helicobacter pylori* 4 weeks or longer after the end of therapy [29]
IBS	Improvement in IBS clinical scores, such as:
	• IBS-related symptom score [30]
	• Functional Bowel Disorder Severity Index [31]
	• IBS Severity Scoring System [32]
IBD - Crohn’s disease	Improvement in the Crohn's Disease Activity Index [33]
IBD - ulcerative colitis	Improvement in the clinical scores, such as Mayo Clinic Score [34], Modified Mayo Disease Activity Index [35]
GERD	Improvement in clinical scores, such as GERD score [36], Gastroesophageal Reflux Disease Activity Index [37]
GERD, gastroesophageal reflux disease; IBD, inflammatory bowel disease; IBS, irritable bowel disease.	

This systematic review will not impose any restrictions on publication status, time period, language of dissemination, and duration of follow-up. We will exclude animal studies.

### Information sources

We will search the following electronic databases from inception to 31 August 2013 using medical subject headings (MeSH) and text words related to turmeric and digestive disorders: MEDLINE, EMBASE, AMED, ProQuest Dissertations & Theses Database, and the Cochrane Central Register of Control Trials using a search strategy (Additional file 1: Appendix 1) drafted by an experienced librarian. We will also search for grey literature through drug regulatory agencies (that is, the National Center for Complementary and Alternative Medicine (NCCAM), European Medicines Agency (EMEA)), Comprehensive Database of Natural Medicines, trial registry website (http://www.clinicaltrials.gov), and abstracts/proceedings from conferences, such as the World Congress of Gastroenterology, the International Society for Pharmacoeconomics and Outcomes Annual International Meeting, and the Annual Cochrane Colloquium. The reference lists of the studies included in the review will also be searched. Moreover, experts in the field will be contacted to identify relevant studies.

### Study selection process

Study selection process consists of three levels. For level 1, titles and abstracts identified from the literature search will be screened independently by the review team. We will conduct a calibration exercise using a random sample of 50 citations that will be screened by all team members independently to identify those that meet eligibility criteria. Inter-rater agreement for study inclusion will be calculated using the percent agreement, whereby the study screening process will continue only if >90% of agreement is observed. If there is any discrepancy (that is, <90% agreement), it will be resolved through a team discussion. A second exercise with another 50 articles will be conducted if poor agreement is noted. A similar process will be repeated for screening the full-text of the relevant articles (level 2) to determine if they meet eligibility criteria. For the data abstraction process (level 3), a random sample of five included studies will be piloted. Similarly, the abstraction process will start only if the percent agreement is greater than 90%. In this phase, the reviewers will independently extract data, including study characteristics (study design, year of study conduct, duration of the study, setting, sample size, country of study conduct), patient characteristics (number of patients, mean age and standard deviation, type of digestive disorders, severity of digestive disorders, place of residences (hospital or community settings), history of digestive disorders, use of gastrointestinal drugs, use of non-steroidal anti-inflammatory drugs (NSAIDs), and co-morbidities), description of intervention and comparators (number of arms, sample size for each group, turmeric species, type of turmeric preparations, dose, duration of use, frequency of use), and study outcomes (proportion of patients reporting treatment success, prevalence of individual symptom associated with digestive disorders, proportion of patients who experienced relapse, the number of physician and hospitalization visits due to digestive disorders, disease-specific quality of life, and general health-related quality of life).

### Methodological quality/risk of bias appraisal

Assessment of the risk of bias pertaining to the included studies will be performed independently by all reviewers, whereby the risk of bias tools will be used in accordance with the study design. Risk of bias for RCTs as well as non-RCTs, such as controlled before-and-after and interrupted time series studies, will be assessed using the nine-item checklist suggested by the Effective Practice and Organization of Care group [38]. This tool assesses the following domains of bias: sequence generation, allocation concealment, baseline outcome measurement, baseline characteristic measurement, incompleteness outcome data, blinding of outcome assessment, contamination, selective outcome reporting, and other types of bias. For observational studies, we will appraise the methodological quality using the nine-item Newcastle-Ottawa Scale [39]. The quality of case–control studies will be assessed according to adequate definition of cases, representativeness of cases, selection of controls, definition of controls, comparability of cases and controls on the basis of the design or analysis, exposure assessment, same method of ascertainment for all subjects, and non-response rate. The quality of cohort studies will be assessed on the basis of representativeness of the exposed cohort, selection of the unexposed cohort, ascertainment of exposure, demonstration that outcome of interest was not presented at start of study, comparability of cohorts on the basis of the design or analysis, outcome assessment, sufficient follow-up duration to capture outcomes, adequacy of follow-up of cohorts. Disagreement will be resolved by a team discussion. To ascertain that we have sufficient power to distinguish chance from real symmetry, the potential for publication bias will be examined using funnel plots only when at least 10 studies are included in the meta-analysis [40].

### Synthesis of included studies

We will first describe study characteristics, patient characteristics, risk of bias/methodological quality results, and summarize the reported outcomes of the included studies. If the data abstraction process yields sufficient data (that is, at least three studies), and the included studies are adequately similar in terms of participants, type of digestive disorders, interventions, outcome measures, study designs, and risk of bias, each specified review outcome will be pooled using a random-effect meta-analysis. Summary statistics for continuous outcomes will be expressed as mean difference and standardized mean difference with 95% CIs. Mean difference will be estimated if the included studies reported the outcomes using the same scale. Standardized mean difference will be estimated if the included studies presented outcomes using different scales - for example, the use of Functional Bowel Disorder Severity Index and IBS Severity Scoring System to represent the severity of IBS. For dichotomous data, summary statistics will be expressed as a risk ratio with 95% CI for ease of interpretation. These analyses will be performed using SAS version 9.3 (SAS Institute, Cary NC).

Clinical heterogeneity or variability in the participants, the types of outcome measurements, and intervention characteristics will be assessed by clinical experts in the field and a research team. Methodological heterogeneity of the included studies will be assessed by a team discussion, while heterogeneity of the summary treatment effects (statistical heterogeneity) will be evaluated using I^2^ statistics. If extensive clinical or methodological heterogeneity is observed, or substantial statistical heterogeneity (that is, I^2^ statistic ≥60%) is identified, we will attempt to explain the underlying causes of heterogeneity. In particular, if the number of included studies permit, we will undertake subgroup analyses based on: 1) age (≤18 years old vs >18 years old); 2) dose of turmeric; 3) concomitant use of NSAIDs; 4) place of residence (hospitals vs community settings); and 5) turmeric species (such as *Curcuma domestica*, *Curcuma longa*). If a pre-specified subgroup analysis is not feasible due to a small number of included studies, we will use narrative synthesis and present outcomes by type of GI condition, type of intervention, study design, and type of outcome.

In cases of missing data, we plan to contact investigators or study sponsors. A series of sensitivity analyses will be also conducted to assess the impact of the following factors on the main finding: dosage and formulation of turmeric, patient attrition rate, and type of study.

## Discussion

This systematic review has significant clinical implication. In particular, if the gastroprotective effect of turmeric is confirmed, turmeric and its compounds should be considered as a promising alternative for patients who suffer from digestive disorders because it is safe [41], inexpensive, and ubiquitously available. In contrast, if gastroprotective effects of turmeric cannot be established, this evidence would be useful in enhancing the patients’ understanding of the limitations of this commonly used therapy. Thus, it could guard them against the false claims and advertisements of turmeric product manufacturers.

To ensure that our findings have clinical impact on patients with digestive disorders, we plan to disseminate our findings by presenting at relevant conferences, publishing in a peer-reviewed journal as well as newspapers, newsletters, and websites of interested organizations.

## Abbreviations

GERD: gastroesophageal reflux disease; GI: gastrointestinal; IBD: inflammatory bowel disease; IBS: irritable bowel disease; NSAID: non-steroidal anti-inflammatory drug; RCT: randomized controlled trial; UC: ulcerative colitis.

## Competing interests

The authors declare that they have no competing interests.

## Authors’ contributions

KT formulated the research question and registered the protocol with the PROSPERO database. KT, MMM, and SES designed the study. MMM obtained the funding for the study. All authors drafted, revised and approved the final protocol.

## Supplementary Material

Additional file 1 **Appendix 1.** Draft search strategy. Click here for file
