# Supplementary material for: Efficacy of turmeric in the treatment of digestive disorders: a systematic review and meta-analysis protocol
Source: Syst Rev. 2014 Jun 28;3:71. doi: 10.1186/2046-4053-3-71 (PMC4080703; doi:10.1186/2046-4053-3-71)
Supplement: Additional file 1 — Appendix 1. Draft search strategy. [file 2046-4053-3-71-S1.docx]

**APPENDIX 1: DRAFT SEARCH STRATEGY**

1 Dyspepsia/ [digestive disorder terms ] (7307)

2 dyspepsia.mp. (11241)

3 (epigastric adj2 pain).tw. (3219)

4 (epigastric adj2 burn$).tw. (95)

5 Peptic Ulcer/ (27642)

6 Duodenal Ulcer/ (23000)

7 Stomach Ulcer/ (23742)

8 (pep$ adj2 ulcer$).tw. (22424)

9 (stomach adj2 ulcer$).tw. (1489)

10 (duoden$ adj2 ulcer$).tw. (19291)

11 (gastr$ adj2 ulcer$).tw. (20676)

12 (marginal adj2 ulcer$).tw. (301)

13 (curling$ adj2 ulcer$).tw. (65)

14 (bleed$ adj2 ulcer$).tw. (3719)

15 (rebleed$ adj2 ulcer$).tw. (95)

16 (re-bleed$ adj2 ulcer$).tw. (13)

17 exp Inflammatory Bowel Diseases/ (59865)

18 Enterocolitis/ (1519)

19 exp Proctitis/ (2345)

20 Ileitis/ (1722)

21 crohn$.tw. (33151)

22 colit$.tw. (45217)

23 ileitis.tw. (1721)

24 enterocolit$.tw. (13289)

25 entero-colit$.tw. (39)

26 proctocolit$.tw. (333)

27 procto-colit$.tw. (11)

28 "inflamm$ bowel$ disease$".tw. (27263)

29 Irritable Bowel Syndrome/ (4218)

30 (colonic adj disease$).tw. (968)

31 (colon adj disease$).tw. (188)

32 "irritable bowel syndrome$".tw. (8084)

33 (irritable adj bowel$).tw. (8348)

34 (irritable adj colon$).tw. (402)

35 (functional adj bowel$).tw. (740)

36 (functional adj colon$).tw. (79)

37 (spastic adj bowel$).tw. (4)

38 (spastic adj colon$).tw. (54)

39 exp Gastroesophageal Reflux/ (22057)

40 (gastroesophageal adj reflux).tw. (13672)

41 (gastro-esophageal adj reflux).tw. (1099)

42 GERD.tw. (5610)

43 (gastro-oesophageal adj reflux).tw. (3756)

44 "gastric acid reflux".tw. (40)

45 Heartburn/ (1728)

46 Esophagitis, Peptic/ (4556)

47 (peptic adj esophagitis).tw. (249)

48 (reflux adj esophagitis).tw. (2750)

49 (digestive adj disorder$).tw. (926)

50 (gastrointestinal adj bleed$).tw. (11809)

51 (gastro-intestinal adj bleed$).tw. (379)

52 (gastrointestinal adj hemorrhag$).tw. (3831)

53 (gastro-intestinal adj hemorrhag$).tw. (88)

54 (gastrointestinal adj haemorrhag$).tw. (1100)

55 (gastro-intestinal adj haemorrhag$).tw. (134)

56 or/1-55 (243670)

57 Curcuma/ [ turmeric terms ] (944)

58 curcum$.tw. (7196)

59 turmeric$.tw. (1478)

60 zedoaria$.tw. (106)

61 Curcumin/ (4828)

62 diferuloylmethane.tw. (274)

63 gelbwurz.tw. (0)

64 halada$.tw. (7)

65 haldi.tw. (27)

66 haridra.tw. (10)

67 (indian adj saffron$).tw. (3)

68 merita.tw. (0)

69 nisha.tw. (4)

70 "pian jiang huang".tw. (1)

71 rajani.tw. (4)

72 safran.tw. (62)

73 souchet.tw. (1)

74 (yu adj jin).tw. (3)

75 or/57-74 (7882)

76 56 and 75 (175)

77 exp Animals/ not (exp Animals/ and Humans/) (4029400)

78 76 not 77 (105)
